# Serological Diagnostics of Lyme Borreliosis: Comparison of Universal and Borrelia Species-Specific Tests Based on Whole-Cell and Recombinant Antigens

**DOI:** 10.1128/JCM.00601-18

**Published:** 2018-10-25

**Authors:** Petr Kodym, Zuzana Kurzová, Dagmar Berenová, Dušan Pícha, Dita Smíšková, Lenka Moravcová, Marek Malý

**Affiliations:** a Department of Zoonoses with Natural Focality, Centre for Epidemiology and Microbiology, National Institute of Public Health, Prague, Czech Republic; b Department of Infectious Diseases, 2nd Faculty of Medicine, Charles University and Na Bulovce Hospital, Prague, Czech Republic; c Department of Biostatistics, National Institute of Public Health, Prague, Czech Republic; University of Tennessee at Knoxville

**Keywords:** Borrelia burgdorferi, Lyme disease, comparative studies, immunoassays, serology

## Abstract

The study compares diagnostic parameters of different commercial serological kits based on three different antigen types and correlates test results with the status of the patient's Borrelia infection. In total, 8 IgM and 8 IgG kits were tested, as follows: enzyme-linked immunosorbent assay (ELISA) (Euroimmun) based on whole-cell antigen, 3 species-specific enzyme immunoassays (EIAs) (TestLine), Liaison chemiluminescence (DiaSorin), ELISA-Viditest (Vidia), EIA, and Blot-Line (TestLine) using recombinant antigens. All tests were performed on a panel of 90 samples from patients with clinically characterized borreliosis (53 with neuroborreliosis, 32 with erythema migrans, and 5 with arthritis) plus 70 controls from blood donors and syphilis patients. ELISA based on whole-cell antigens has superior sensitivity and superior negative predictive value and serves as an excellent screening test, although its specificity and positive predictive values are low. Species-specific tests have volatile parameters. Their low sensitivity and low negative predictive value handicap them in routine diagnostics. Tests with recombinant antigens are characterized by high specificity and high positive predictive value and have a wide range of use in diagnostic practice. Diagnostic parameters of individual tests depend on the composition of the sample panel. Only a small proportion of contradictory samples giving both negative and positive results is responsible for discrepancies between test results. Correlation of test results with the patient's clinical state is limited, especially in the erythema migrans group with high proportions of negative and contradictory results. In contrast, IgG test results in the neuroborreliosis group, which are more concordant, show acceptable agreement with Borrelia status.

## Abstract (precis)

The study compares diagnostic parameters of different commercial serological kits based on three different antigen types and correlates test results with the status of the patient's Borrelia infection. In total, 8 IgM and 8 IgG kits were tested, as follows: enzyme-linked immunosorbent assay (ELISA) (Euroimmun) based on whole-cell antigen, 3 species-specific enzyme immunoassays (EIAs) (TestLine), Liaison chemiluminescence (DiaSorin), ELISA-Viditest (Vidia), EIA, and Blot-Line (TestLine) using recombinant antigens.

## INTRODUCTION

Lyme borreliosis, the most abundant tick-borne human disease in the world, is caused by spirochetes of the Borrelia burgdorferi sensu lato complex (1). During the course of infection, a skin lesion can appear after a tick bite, and clinical manifestations of this multisystemic disease (which comprise musculoskeletal, cardiovascular and nervous system involvement, subsequent arthritis, and skin damage or chronic central nervous system [CNS] inflammation) vary widely, most often manifesting with a variety of signs and symptoms, such as erythema migrans (EM), neuroborreliosis, and Lyme arthritis (2). The case definitions for European Lyme borreliosis emphasize the recognition of clinical manifestations supported by relevant laboratory criteria (3). Because clinical signs are not always unmistakable and since other laboratory methods, such as PCR, play only an auxiliary role according to the majority of diagnostic guidelines (3–6), a diagnosis of “Lyme borreliosis” is often based on serology tests.

Hand in hand with improved antigen composition and the introduction of more sophisticated systems, the accuracy of serological testing has significantly increased over the last 2 decades, and accuracy improvement by combining more tests, like two-tiered testing, is recommended by most guidelines. However, the basic problem of serological diagnostics of Lyme borreliosis, nonexistence of a gold standard, makes it virtually impossible to standardize diagnostic tools and find a reliable correlation between the results of serological tests and the clinical status of a patient. There is a full spectrum of different variants of immunoenzymatic and analogical tests (e.g., chemiluminescent tests) appearing on the market, as well as different immunoblot assays using whole-cell or recombinant antigens of different species of Borrelia burgdorferi sensu lato, whose objective diagnostic parameters are unknown. Without information on the relative sensitivity of the test used, results cannot be adequately interpreted. Antigenic heterogeneity of the Borrelia burgdorferi sensu lato complex can also complicate the situation; inappropriate species or even geographic origin of the antigen used could be the cause of false-negative results in some samples (7–9). As a result of these or other difficulties, the predictive value of serological tests is far from optimal, leading to incorrect diagnosis in some cases. Inadequate interpretation can result not only in superfluous antibiotic therapies targeting Lyme disease but can also fail to solve the patient's problems (10), whereas false-seronegative patients can remain untreated.

The aims of the present study are to compare diagnostic parameters of different commercial serological kits based on three different antigen types (recombinant, whole-cell Borrelia species mixture, and species-specific whole cell) and to correlate the test results with the status of the patient's Borrelia infection. These data are of great importance for pertinent selection of serological method appropriate for a required examination and correct interpretation of serological test results.

## MATERIALS AND METHODS

The study was approved by the local Ethics Committees of Na Bulovce Hospital and University Hospital Kralovske Vinohrady, Prague, Czech Republic, and was conducted in accordance with the ethical standards laid down by the 1975 Declaration of Helsinki, as revised in 2008.

### Examined samples.

Diagnostic methods were tested on serum and plasma specimens from patients and controls grouped according to clinical criteria, as follows:

### Borreliosis group.

Borreliosis group specimens (*n* = 90) were collected from patients of the Na Bulovce Hospital Infectious Diseases Clinic in Prague, Czech Republic, with recent clinical symptoms of borreliosis manifested as erythema migrans (EM) (*n* = 32), Lyme arthritis (LA) (*n* = 5), or neuroborreliosis (NB) (*n* = 53). EM diagnosis was established by the clinical picture (typical rash of >5 cm in diameter), a history of tick bite, and a delay in appearance of symptoms of at least 2 days following a tick bite. Antibody testing was performed 33 to 112 days after initiation of treatment. Neuroborreliosis criteria were as follows: (i) lymphocyte pleocytosis of >5 cells/mm^3^, (ii) intrathecal synthesis of anti-Borrelia antibodies in cerebrospinal fluid (CSF) (antibody index CSF/serum), and (iii) clinical symptoms compatible with neuroborreliosis (3). Only patients completely fulfilling all of the criteria were included in this group. Patients with joint swelling and antibody or PCR positivity in synovial fluid (3 patients) or other current clinical manifestations typical for Lyme borreliosis (LB) (EM and/or NB, 2 patients) were included in the Lyme arthritis group.

### Control group.

Control group (*n* = 70) samples were obtained from persons with no clinical signs of Lyme borreliosis, comprising samples from healthy blood donors (BD) (*n* = 60) at the Transfusion Centre of the University Hospital Kralovske Vinohrady, Prague, and from patients with serologically confirmed syphilis (CS) (*n* = 10), supplied by the National Reference Laboratory for the Diagnostics of Syphilis (The National Institute of Public Health, Prague, Czech Republic).

All samples supplied to the laboratory were anonymized. All patients agreed to participate in the study and signed an informed consent form.

### Serological methods.

A total of 16 serological tests from four manufacturers were used in determining antibodies against Borrelia burgdorferi sensu lato, namely 8 tests for determining specific IgM and 8 for determining IgG (Table 1). The primary criterion in selecting tests for comparison was the type of antigen on which is the test was based. As representatives of recombinant and whole-cell antigen methods (not the species-specific ones), particular tests used frequently in Central Europe were chosen. Tests were carried out and evaluated exactly according to the manufacturer's instructions.

**TABLE 1:** Characteristics of 8 compared tests for the detection of IgM or IgG anti-Borrelia antibodies

Method^*a*^	Type of assay^*b*^	Kit, manufacturer^*c*^	Antigens^*d*^
Whole-cell antigens			
WEE-IgM/IgG	ELISA	Anti-Borrelia ELISA (IgM)/Anti-Borrelia plus VlsE ELISA (IgG), Euroimmun	Antigen extracts of Borrelia burgdorferi sensu stricto, Borrelia afzelii, and Borrelia garinii; IgG enriched with recombinant VlsE of Borrelia burgdorferi
WSA-IgM/IgG	EIA species-specific	EIA Borrelia afzelii IgM/EIA Borrelia afzelii VlsE IgG, TestLine	Sonicated whole-cell antigen of Borrelia afzelii with high content of p83, p41 (flagellin), p39, OspA, OspC, p18 and p14; IgG enriched with VlsE
WSB-IgM/IgG	EIA species-specific	EIA Borrelia burgdorferi sensu stricto IgM/EIA Borrelia burgdorferi sensu stricto VlsE IgG, TestLine	Sonicated whole-cell antigen of Borrelia burgdorferi sensu stricto with high content of p83, p41 (flagellin), p39, OspA, OspC, p18 and p14; IgG enriched with VlsE
WSG-IgM/IgG	EIA species-specific	EIA Borrelia garinii IgM/EIA Borrelia garinii VlsE IgG, TestLine	Sonicated whole-cell antigen of Borrelia garini with high content of p83, p41 (flagellin), p39, OspA, OspC, p18 and p14; IgG enriched with VlsE
Recombinant antigens			
RCL-IgM/IgG	CLIA chemiluminescence immunoassay	Liaison Borrelia IgM II/IgG, DiaSorin	Recombinant VisE; IgM enriched with OspC
RET-IgM/IgG	EIA	EIA Borrelia recombinant IgM/IgG, TestLine	Combination of selected parts of specific antigens, as follows: OspC, internal flagelin-p41 a p39 of Borrelia burgdorferi sensu lato species; IgG enriched with p83 and p17 (IgG)
REV-IgM/IgG	ELISA	ELISA-Viditest anti-Borrelia recombinant IgM/IgG (CSF), Vidia	Mixture of specific recombinant antigens
RBT-IgM/IgG	Blot-Line	Blot-Line Borrelia/HGA IgM, TestLine	Recombinant antigens, as follows: p41 Ba, p39 Ba, OspC Ba, OspC Bg, OspC Bs, and p44
		Blot-Line Borrelia/HGA IgG, TestLine	Recombinant antigens, as follows: VlsE Ba, VlsE Bg, VlsE Bs, p83 Ba, p41 Ba, p39 Ba, OspB Bs, OspA Ba, OspA Bg, OspA Bs, OspC Bg, p17 Bg, p44, and TpN17

a WEE, ELISA based on a whole-cell antigen (Euroimmun); WSA, ELISA based on a whole-cell antigen, specific to B. afzelii; WSB, ELISA based on a whole-cell antigen, specific to B. burgdorferi; WSG, ELISA based on a whole-cell antigen, specific to B. garinii; RCL, CLIA based on a recombinant antigen (DiaSorin); RET, EIA based on a recombinant antigen (TestLine); REV, ELISA based on a recombinant (Vidia); RBT, Blot-Line test based on a recombinant antigen (TestLine).

b ELISA, enzyme-linked immunosorbent assay; EIA, enzyme immunoassay; and CLIA, chemiluminescent immunoassay.

c HGA, human granulocytic anaplasmosis.

d The data on the antigens used were taken from the manufacturer's instructions.

### Statistical methods.

The outcome of individual diagnostic methods is presented as percentages of positive and borderline results in individual groups. Agreement between methods is expressed as follows. When all 8 tests (IgM or IgG) yielded a congruent positive/negative result, this was defined as “unanimously positive/negative.” If a given sample showed negative or borderline test results via different methods, the result was defined as broadly negative. In the case of positive or borderline results, the result was expressed as broadly positive. Samples in which 3 to 5 results were positive and the remainder were negative or borderline were defined as contradictory.

Statistical comparison of the concordance of individual serological test results was based on Bowker's test of symmetry in the contingency table with three categories (negative, borderline, and positive). Comparison was made for all test pairs. Due to the low frequencies in some categories, the exact *P* values were computed. *P* values of less than 0.05 were considered statistically significant.

To evaluate the predictive value of individual diagnostic methods, the results of serum evaluation were compared with the clinical classification of persons from whom the serum was obtained. For this purpose, the borderline results of serological tests were included with the positive ones. The results are characterized by the sensitivity, specificity, and predictive values of the positive and negative tests.

Stata 9.2 (StataCorp LP, College Station, TX) statistical software was used for evaluation.

## RESULTS

Results from tests used for samples taken from an individual group of patients with Lyme borreliosis and the control group are shown in Tables 2 and 3. The distribution of positivity rates is considerably variable. Consequently, the proportion of negative results differs substantially among groups; in blood donors the ranges were 71.1 to 100.0% and 68.3 to 95.0% for IgM and IgG tests, respectively. The respective ranges in the neuroborreliosis group were 18.9 to 52.8% and 11.3 to 20.8%, and for the EM group, 31.3 to 75.0% and 37.5 to 71.9%, respectively.

**TABLE 2:** Positivity rates for the panel of samples from patients with Lyme borreliosis and controls examined by compared tests for the detection of anti-Borrelia IgM

IgM test	Positivity rate (%):								
	Controls (*n* = 70)		Lyme borreliosis (*n* = 90)			Controls, all categories (*n* = 70)		Borreliosis, all categories (*n* = 90)	
	Blood donors (*n* = 60), positive	Syphilis (*n* = 10), positive	Neuroborreliosis (*n* = 53), positive	EM (*n* = 32), positive	Lyme arthritis (*n* = 5), positive	Borderline	Positive	Borderline	Positive
WEE	15.0	30.0	66.0	53.1	60.0	11.4	17.1	5.6	61.1
WSA	3.3	10.0	62.3	43.8	60.0	1.4	4.3	2.2	55.6
WSB	0.0	10.0	41.5	18.8	0.0	0.0	1.4	5.6	31.1
WSG	3.3	10.0	58.5	37.5	20.0	5.7	4.3	6.7	48.9
RCL	10.0	20.0	49.1	31.3	0.0	1.4	11.4	3.3	40.0
RET	1.7	20.0	64.2	43.8	40.0	2.9	4.3	5.6	55.6
REV	0.0	30.0	71.7	46.9	80.0	2.9	4.3	5.6	63.3
RBT	1.7	30.0	54.7	50.0	20.0	18.6	5.7	22.2	51.1

**TABLE 3:** Positivity rates for the panel of samples from patients with Lyme borreliosis and controls examined by compared tests for the detection of anti-Borrelia IgG

IgG tests	Positivity rate (%) for:								
	Controls (*n* = 70)		Lyme borreliosis (*n* = 90)			Controls, all categories (*n* = 70)		Borreliosis, all categories (*n* = 90)	
	Blood donors (*n* = 60), positive	Syphilis (*n* = 10), positive	Neuroborreliosis (*n* = 53), positive	EM (*n* = 32), positive	Lyme arthritis (*n* = 5), positive	Borderline	Positive	Borderline	Positiveosit
WEE	16.7	40.0	86.8	46.9	40.0	17.7	20.0	6.7	70.0
WSA	18.3	30.0	77.4	50.0	40.0	2.9	20.0	3.3	65.6
WSB	3.3	30.0	77.4	34.4	20.0	1.4	7.1	6.7	58.9
WSG	1.7	20.0	79.0	18.8	20.0	5.7	4.3	3.3	54.4
RCL	8.3	20.0	79.3	37.5	60.0	7.1	10.0	3.3	63.3
RET	3.3	10.0	81.1	28.1	20.0	1.4	4.3	3.3	58.9
REV	15.0	20.0	81.1	37.5	40.0	1.4	15.7	2.2	63.3
RBT	1.7	10.0	67.9	43.8	40.0	10.0	2.9	18.9	57.8

Qualitative results of examinations by all compared tests correlated in the majority of samples. Taking together all (both unanimously and broadly) negative and positive IgM test samples, we find general concordance in 89 (55.6%) samples for IgM and in 107 (66.9%) samples for IgG. For IgM and IgG, 44 (27.5%) samples reacted negatively, of which 29 (18.1%) samples had unanimous test concordance and 15 (9.4%) samples had broad test concordance. Only in 19 (11.9% for IgM) and 17 (11.6% for IgG) cases were the individual tests contradictory. The highest number of contradictory IgM test samples was in the neuroborreliosis group (20.7%) and the highest number of IgG contradictory IgG test samples was in the erythema migrans group (28.2%) (see Table S3 in the supplemental material).

Tests based on different principles and using different antigens reacted differently to the same samples. Bowker's test of symmetry showed prevailing conformity between various tests with recombinant antigens, both in IgM and IgG tests; enzyme-linked immunosorbent assay (ELISA) with whole-cell antigens was also in agreement with almost all tests. On the other hand, most species-specific enzyme immunoassays (EIAs) were statistically different from all other tests. These conformities and differences go hand in hand with parameters such as sensitivity, specificity, and predictive values which characterize test performance (see Tables S1, S2, and S3 in the supplemental material). In the highly sensitive ELISA method, using whole-cell antigen (WEE), a high percentage of samples in the borreliosis group was positive or borderline in anti-borrelial IgM and/or IgG tests. The sensitivity and predictive values of a negative test are high. However, a large number of control samples tested positive, and thus the specificity and positive predictive values are low (Tables S1, S2, and S3).

The opposite is true of some species-specific tests, primarily WSB-IgM and WSG-IgG tests, which have low sensitivity. When a sample is captured by such a test, the response is highly specific, and the predictive value of the positive test is high, whereas the negative test has a low predictive value.

Tests based on recombinant antigens, such as those with an immunoenzymatic or chemiluminescence base, are sufficiently sensitive and at the same time achieve high specificity while the predictive values of negative and positive tests are similar. Blot RBT-IgG has such balanced parameters, whereas the parameters of the RBT-IgM test are closer to the characteristics of a sensitive method. A certain balance between sensitivity and specificity is seen in some species-specific EIAs, primarily the WSG-IgM. If all three species-specific tests, (WSA, WSB, and WSG) were evaluated in summary, the result was considered positive if at least one of the tests was positive. Data show that their sensitivity approached that of methods using recombinant antigens, albeit with somewhat lower specificity (data not shown). The characteristics of the borrelial IgM tests come out generally worse in comparison with those of IgG tests. The sensitivity, specificity, and predictive values of individual IgG tests are far more balanced and there are usually markedly smaller intertest differences. In the neuroborreliosis group, higher sensitivity was achieved in IgG tests, and, by contrast, specificity was higher for IgM tests. Likewise, predictive values of negative and positive IgM and IgG tests depend more on the group of samples than on the serological test used. For the neuroborreliosis group, the characteristics are more favorable than in the problematic EM group. In this group, the characteristics of individual tests are the least balanced and without a clear conclusion in relation to classes of IgG and IgM antibodies.

## DISCUSSION

Due to the lack of a serological gold standard, our study evaluated parameters of all 8 tests on the basis of samples taken from patients with clinically manifested borreliosis (where a positive reaction is expected) and of control samples (where a negative result is anticipated). According to the comparison of the concordance of individual serological test results based on Bowker's test of symmetry and in accordance with the sensitivity, specificity, and predictive values of negative and positive tests, it is possible to divide these tests into four groups, which differ in the following attributes and possibilities of application.

Immunoenzymatic tests based on a mixture of whole-cell antigens (EUROIMMUN) have high sensitivity and predictive value for negative tests detecting anti-borrelial IgG and IgM, and statistical agreement with other tests is high. On the other hand, their specificity and positive predictive value are low. Quite a high percentage of these results are borderline, reducing the predictive value of the test. Whole-cell ELISA serves as an excellent screening test, but must be confirmed by specific methods, usually immunoblotting.

Immunoenzymatic tests based on whole-cell species-specific antigens of Borrelia afzelii, Borrelia garinii, and B. burgdorferi (TestLine) have widely differing parameters and low statistical agreement with other tests. Generally, they have low sensitivity and low negative predictive value, whereas specificity and predictive values of positive tests are high. Because of their very low sensitivity, they are unsuitable even as confirmation methods. Sensitivity increases when all three species-specific tests are performed simultaneously, and a positive result from at least one of them counts for summarizing positivity. The inconveniences of this procedure are reflected by the necessity of performing each test three times and by the deteriorating specificity and predictive values of the positive test. The fact that individual species-specific tests give different results for different categories of patients favors them for research use. Not even serotyping of Borrelia in patients with the help of species-specific immunoenzymatic methods or Western blotting is possible. For common, routine diagnosis of Lyme borreliosis, species-specific serological tests are not beneficial.

Blot-Line with recombinant antigens (TestLine) reaches high sensitivity and negative predictive value, even where the specificity and predictive value of positive tests is high. The disadvantage of this assay is a high percentage of hard-to-interpret borderline results.

Immunoenzymatic (TestLine and Vidia) and chemiluminescence (DiaSorin) tests with recombinant antigens are characterized by a combination of high specificity and predictive value of the positive test. According to Bowker's test of symmetry, there is no statistically significant difference in results from these methods, which have a wide range of use in diagnostic practice.

Some of the compared tests, like Liaison chemiluminescence (Diasorin) (11, 12), ELISA by Euroimmun (11–13), and EIA Borrelia garinii and EIA Borrelia recombinant IgM and IgG (TestLine) (14) have already been evaluated. According to published studies, serological tests based on a whole-cell antigen (15, 16) or on detergent extracts from Borrelia (17) came out as the most sensitive but were less specific. On the contrary, systems based on recombinant antigens showed the highest specificity, along with low but acceptable sensitivity (16–18). On the other hand, Ang et al. (13) could not find clear relationships between assay and the fraction of positive tests or between the specificity and the nature of the antigen used for serological tests. The low number of assays using each type of antigen limits the scope of the present study.

Resulting sensitivity and specificity values and other characteristics of individual tests are directly dependent on the composition of the comparison sample panel. Most samples show concordant results in all 8 compared tests. On the other hand, only a small proportion of samples (1/8 of panels in IgM tests and 1/10 in IgG tests) giving contradictory results is responsible for discrepancies. If the problematic samples were removed from the panel, the sensitivity and specificity would be balanced and hence closer to 100% in all tests, as claimed by some manufacturers in their promotional material. However, in real-life diagnostic practice it is necessary to examine real samples, including problematic ones, and so ideal parameters cannot be reached using any method.

The most problematic group was erythema migrans, which had the highest percentage of contradictory and negative samples (15.6% samples unanimously or broadly negative for IgM and IgG) and a low test sensitivity, fluctuating between 25.0% and 68.8% for IgM and 28.1% to 62.5% for IgG. It is evident that not every patient with EM will necessarily produce anti-borrelial IgM and IgG, as expected in the sensitivity calculation, and the predictive value of both negative and positive tests provides the worst results. Similarly poor test parameters, also indicated by other authors (18–20), are the reason why detection of antibodies is not an ideal method for EM diagnostics (3).

On the contrary, the tests were far more successful in diagnosing neuroborreliosis. The sensitivity of individual tests ranged from 47.2% to 81.1% for IgM and 79.2% to 88.7% for IgG; 69.8% of samples were unanimously or broadly IgG positive, no unanimously negative samples for IgM and IgG were seen (Tables S1 and S2). This confirms a relatively reliable production of antibodies, particularly of IgG, in neuroborreliosis.

Meta-analysis (20) reveals that the average sensitivity of a serological test is the lowest during the erythema migrans stage of infection (46.5% of seropositive patients), whereas in neuroborreliosis the percentage of seropositive patients increased up to 87.3% and in Lyme arthritis IgG it increased up to 95.8%. The contrasting low proportion of seropositive patients in our Lyme arthritis group could be due to the low number of included patients; two patients with recent EM refused examination of synovial fluid, so their diagnosis remained highly probable only.

Blood donors were 71.7 to 100.0% negative for IgM and 68.3 to 95.0% negative for IgG, whereas 53.3% of samples contained no anti-Borrelia antibodies (or gave borderline values only) in any IgM or IgG test. When samples in this group are positive, they are frequently discordant but never unanimously or broadly positive. It appears that the cause of sample positivity was nonspecific reactivity rather than ongoing borreliosis or remainder antibodies persisting after previous clinical or subclinical infection (21). Nonetheless, the possibility cannot be excluded that some of the results presented here as false positive could be the consequence of infection. The presence of specific antibodies does not prove the presence of disease. Specific antibodies usually fade away over a period of months (IgM) or a few years (IgG) but may persist for 10 to 20 years. Moreover, a relatively high background seroprevalence of both specific IgG and IgM can be found in the healthy population of an area where Borrelia is endemic (22).

Another cause of false-positive results could be cross-reactivity of the serological test. Some studies have indicated that Epstein-Barr virus and cytomegalovirus are the main causes of false-positive reactions in IgM EIAs (23). It is possible that some tests are cross-reacting with anti-Treponema antibodies. Each method declared 1 to 3 positive reactions in the syphilis group for both classes of antibodies. Although only 3 of 10 samples were absolutely negative (and it cannot be excluded or confirmed that some of the patients of this group really underwent Lyme borreliosis), the question concerning cross-reactivity cannot be conclusively answered. Because of low specificity the results of antibody testing can only be interpreted together with clinical data and CSF inflammation parameters. Therefore, antibody testing should only be carried out in patients with symptoms suggestive of Lyme neuroborreliosis (5).

Especially in Europe, where several pathogenic species of the Borrelia burgdorferi sensu lato complex occur, the heterogeneity of the immunodominant epitopes of infecting strains can result in lower test sensitivity if an inappropriate antigen is used (7). Different strains and geographical origins of Borrelia samples that are used for preparation of diagnostic antigens may likewise play a role. The conclusion that the use of a Western blot (WB) analysis with a European strain for detecting Lyme borreliosis will provide higher sensitivity for a European serum panel than a WB with an American isolate (8, 9) was not confirmed (23).

Our pilot study did not confirm the hypothesis that serological test sensitivity is reduced by species and antigen-bound borrelial variability (24). On the contrary, it turned out that species-specific tests are able to detect antibodies against other species of Borrelia. Tests with recombinant antigens and even other tests, including species-specific ones, do not present greater problems with detection of antibodies in patients infected with other species of Borrelia burgdorferi sensu lato than the basic triad. Furthermore, in cases of seronegative results from patients with PCR-confirmed borreliosis, sequencing confirmed infection by the most common species, whereas even species-specific tests were negative (24).

A bigger problem than lower sensitivity is low test specificity, particularly in the case of IgM. In practice, physicians are always worried by positive findings of IgM, which is able to persist in repeated blood samples without formation of IgG for months or years even if there is no onset of borreliosis (25). In our study, this is demonstrated by the considerably high percentage of borderline and positive test results (IgM and IgG) in control groups.

It can be concluded that the selected antigens influence diagnostic test parameters to a considerable degree. Whereas ELISA based on whole-cell antigen mixture has superior sensitivity and negative predictive value, its diagnostic value is limited by low specificity and positive predictive value. Species-specific tests have volatile parameters, and low sensitivity and negative predictive value handicap them in routine diagnostics. In comparison, tests with recombinant antigens characterized by high specificity and positive predictive value have a wide range of use in diagnostic practice.

Diagnostic parameters of individual methods depend on the samples tested. Correlation of test results with a patient's clinical state was only limited in the EM group, while in the neuroborreliosis group the agreement was acceptable.

## Supplementary Material

Supplemental file 1
