# Supplementary material for: Serological Diagnostics of Lyme Borreliosis: Comparison of Universal and Borrelia Species-Specific Tests Based on Whole-Cell and Recombinant Antigens
Source: J Clin Microbiol. 2018 Oct 25;56(11):e00601-18. doi: 10.1128/JCM.00601-18 (PMC6204684; doi:10.1128/JCM.00601-18)
Supplement: Supplemental file 1 [file zjm999096145s1.pdf]

Table S1 Sensitivities, specificities and predictive values of compared IgM tests relating to samples of the borreliosis, neuroborreliosis and EM categories, always including all controls

	Sensitivity (%)<br>95% CI			Specificity (%)<br>95% CI			Positive predictive value (%)<br>95% CI			Negative predictive value (%)<br>95% CI		
IgM test	Borreliosis	Neuro-borreliosis	EM	Borreliosis	Neuro-borreliosis	EM	Borreliosis	Neuro-borreliosis	EM	Borreliosis	Neuro-borreliosis	EM
WEE	66.7<br>56.0-76.3	69.8<br>55.7-81.7	62.5<br>43.7-78.9	71.4<br>59.4-81.6	71.4<br>59.4-81.6	71.4<br>59.4-81.6	75.0<br>64.1-84.0	64.9<br>51.1-77.1	50.0<br>33.8-66.2	62.5<br>51.0-73.1	75.8<br>63.6-85.5	80.7<br>68.6-89.6
WSA	57.8<br>46.9-68.1	66.0<br>51.7-78.5	43.8<br>26.4-62.3	94.3<br>86.0-98.4	94.3<br>86.0-98.4	94.3<br>86.0-98.4	92.9<br>82.7-98.0	89.7<br>75.8-97.1	77.8<br>52.4-93.6	63.5<br>53.5-72.7	78.6<br>68.3-86.8	78.6<br>68.3-86.8
WSB	36.7<br>26.8-47.5	47.2<br>33.3-61.4	25.0<br>11.5-43.4	98.6<br>92.3-100.0	98.6<br>92.3-100.0	98.6<br>92.3-100.0	97.1<br>84.7-99.9	96.2<br>80.4-99.9	88.9<br>51.8-99.7	54.8<br>45.7-63.6	71.1<br>61.1-79.9	74.2<br>64.1-82.7
WSG	55.6<br>44.7-66.0	64.2<br>49.8-76.9	40.6<br>23.7-59.4	90.0<br>80.5-95.9	90.0<br>80.5-95.9	90.0<br>80.5-95.9	87.7<br>76.3-94.9	82.9<br>67.9-92.9	65.0<br>40.8-84.6	61.2<br>51.1-70.6	76.8<br>66.2-85.4	76.8<br>66.2-85.4
RCL	43.3<br>32.9-54.2	54.7<br>40.4-68.4	31.3<br>16.1-50.0	87.1<br>77.0-94.0	87.1<br>77.0-94.0	87.1<br>77.0-94.0	81.3<br>67.4-91.1	76.3<br>59.8-88.6	52.6<br>28.9-75.6	54.5<br>44.8-63.9	71.8<br>61.0-81.0	73.5<br>62.7-82.6
RET	61.1<br>50.3-71.2	71.7<br>57.7-83.2	46.9<br>29.1 - 65.3	92.9<br>84.1-97.6	92.9<br>84.1-97.6	92.9<br>84.1-97.6	91.7<br>81.6-97.2	88.4<br>74.9-96.1	75.0<br>50.9-91.3	65.0<br>54.8-74.3	81.3<br>71.0-89.1	79.3<br>68.9-87.4
REV	68.9<br>58.3-78.2	81.1<br>68.0-90.6	46.9<br>29.1-65.3	92.9<br>84.1-97.6	92.9<br>84.1-97.6	92.9<br>84.1-97.6	92.5<br>83.4-97.5	89.6<br>77.3-96.5	75.0<br>50.9-91.3	69.9<br>59.5-79.0	86.7<br>76.8-93.4	79.3<br>68.9-87.4
RBT	73.3<br>63.0-82.1	77.4<br>63.8-87.7	68.8<br>50.0-83.9	75.7<br>64.0-85.2	75.7<br>64.0-85.2	75.7<br>64.0-85.2	79.5<br>69.2-87.6	70.7<br>57.3-81.9	56.4<br>39.6-72.2	68.8<br>57.3-78.9	81.5<br>70.0-90.1	84.1<br>72.7-92.1

Note: Borderline results are considered positive

95% CI – 95% confidence interval

Table S2 Sensitivity, specificity and predictive values of compared IgG tests relating to samples of the borreliosis, neuroborreliosis and EM categories, always including all controls

IgG test	Sensitivity (%) 95% CI			Specificity (%) 95% CI			Positive predictive value (%) 95% CI			Negative predictive value (%) 95% CI		
	Borreliosis	Neuro-borreliosis	EM	Borreliosis	Neuro-borreliosis	EM	Borreliosis	Neuro-borreliosis	EM	Borreliosis	Neuro-borreliosis	EM
WEE	76.7<br>66.6-84.9	88.7<br>77.0-95.7	59.4<br>40.6-76.3	64.3<br>51.9-75.4	64.3<br>51.9-75.4	64.3<br>51.9-75.4	73.4<br>63.3-82.0	65.3<br>53.1-76.1	43.2<br>28.4-59.0	68.2<br>55.6-79.1	88.2<br>76.1-95.6	77.6<br>64.7-87.5
WSA	68.9<br>58.3-78.2	81.1<br>68.0-90.6	53.1<br>34.7-70.9	77.1<br>65.6-86.3	77.1<br>65.6-86.3	77.1<br>65.6-86.3	79.5<br>68.8-87.8	72.9<br>59.7-83.6	51.5<br>33.5-69.2	65.9<br>54.6-76.0	84.4<br>73.1-92.2	78.3<br>66.7-87.3
WSB	65.6<br>54.8-75.3	79.3<br>65.9-89.2	46.9<br>29.1-65.3	91.4<br>82.3-96.8	91.4<br>82.3-96.8	91.4<br>82.3-96.8	90.8<br>81.0-96.5	87.5<br>74.8-95.3	71.4<br>47.8-88.7	67.4<br>57.0-76.6	85.3<br>75.3-92.4	79.0<br>68.5-87.3
WSG	57.8<br>46.9-68.1	79.3<br>65.9-89.2	28.1<br>13.8-46.8	90.0<br>80.5-95.9	90.0<br>80.5-95.9	90.0<br>80.5-95.9	88.1<br>77.1-95.1	85.7<br>72.8-94.1	56.3<br>29.9-80.3	62.4<br>52.2-71.8	85.1<br>75.0-92.3	73.3<br>62.6-82.2
RCL	66.7<br>56.0-76.3	79.3<br>65.9-89.2	46.9<br>29.1-65.3	82.9<br>72.0-90.8	82.9<br>72.0-90.8	82.9<br>72.0-90.8	83.3<br>72.7-91.1	77.8<br>64.4-88.0	55.6<br>35.3-74.5	65.9<br>55.0-75.7	84.1<br>73.3-91.8	77.3<br>66.2-86.2
RET	62.2<br>51.4-72.2	83.0<br>70.2-91.9	34.4<br>18.6-53.2	94.3<br>86.0-98.4	94.3<br>86.0-98.4	94.3<br>86.0-98.4	93.3<br>83.8-98.2	91.7<br>80.0-97.7	73.3<br>44.9-92.2	66.0<br>55.9-75.2	88.0<br>78.4-94.4	75.9<br>65.5-84.4
REV	65.6<br>54.8-75.3	84.9<br>72.4-93.3	37.5<br>21.1-56.3	82.9<br>72.0-90.8	82.9<br>72.0-90.8	82.9<br>72.0-90.8	83.1<br>72.3-91.0	79.0<br>66.1-88.6	50.0<br>29.1-70.9	65.2<br>54.3-75.0	87.9<br>77.5-94.6	74.4<br>63.2-83.6
RBT	76.7<br>66.6-84.9	86.8<br>74.7-94.5	62.5<br>43.7-78.9	87.1<br>77.0-94.0	87.1<br>77.0-94.0	87.1<br>77.0-94.0	88.5<br>79.2-94.6	83.6<br>71.2-92.2	69.0<br>49.2-84.7	74.4<br>63.6-83.4	89.7<br>79.9-95.8	83.6<br>73.1-91.2

Note: Borderline results are considered positive

95% CI – 95% confidence interval

Table S3 Percentage of samples with concordant and discordant results of eight compared tests in the groups of panel. Unanimously concordant samples show equal qualitative results (positive or negative) in all compared tests, in broadly concordant samples borderline (but not opposite) test results are also acceptable. In contradictory samples, 3-5 tests give positive results and the remainder negative or borderline.

Group	IgM and IgG simultaneously negative samples		IgM positive samples		IgG positive samples		Contradictory samples	
	unanimously %	broadly %	unanimously %	broadly %	unanimously %	broadly %	IgM %	IgG %
Blood donors (N=60)	36.7	16.7	0.0	0.0	0.0	0.0	3.3	8.3
Syphilis (N=10)	30.0	20.0	0.0	0.0	10.0	0.0	0.0	10.0
Neuroboreliosis (N=53)	0.0	3.8	24.5	5.7	52.8	17.0	20.8	3.8
Erythema migrans (N=32)	12.5	3.1	12.5	6.3	15.6	3.1	15.6	28.1
Lyme arthritis (N=5)	0.0	0.0	0.0	0.0	20.0	0.0	20.0	0.0
TOTAL (N=160)	18.1	9.4	10.6	3.1	21.9	6.3	11.9	10.6
